# Informal Social Activities and Cognitive Function in Later Life: The Role of Gender and Marital Status

**DOI:** 10.1093/geroni/igaf122.4145

**Published:** 2025-12-31

**Authors:** Haejin Jang, Sae Hwang Han

**Affiliations:** University of Massachusetts Boston, Boston, Massachusetts, United States; University of Texas at Austin, Austin, Texas, United States

## Abstract

Social engagement with friends and neighbors in informal domains—e.g., having social visits or providing informal helping—is increasingly recognized as a lifestyle factor protective of cognitive function in later life. However, little is known about whether these benefits vary across sociodemographic characteristics, such as gender and marital status. Using eight waves of nationally representative data from the U.S. Health and Retirement Study (person N = 29,777; 131,449 person-wave observations), this study examined how gender and marital status moderated the cognitive outcomes associated with social visits and informal helping. Cognitive function was assessed with the modified Telephone Interview for Cognitive Status (m-TICS), and social visits and informal helping were evaluated as both status and level of involvement (i.e., dose effects). Within-between random effects models were employed to identify within-person effects unbiased by stable person-level characteristics. Findings showed that both forms of social activities were linked to better cognitive function among women, whereas only informal helping predicted better cognitive outcomes among men. Moderate levels of informal helping yielded the greatest benefit for both women and men, while high levels showed diminishing returns; by contrast, only high-frequency social visits were associated with significant cognitive benefits among women. When considering marital status, significant differences appeared only among men, where social visits were linked to better cognition during when men were married, but not when they were unmarried. These findings highlight the importance of tailoring social engagement interventions to reflect differences in gender and marital status, particularly among populations at risk of cognitive decline.

